# The Kinetics of Mesophyll Conductance and Photorespiration During Light Induction

**DOI:** 10.3390/plants14060850

**Published:** 2025-03-08

**Authors:** Ningyu Liu, Jianxin Cao, Mingying Yang, Yiyun Li, Wei Huang

**Affiliations:** 1 Kunming Institute of Botany, Chinese Academy of Sciences, Kunming 650201, China; liuningyu@mail.kib.ac.cn (N.L.); yangmingying@mail.kib.ac.cn (M.Y.); liyiyun@mail.kib.ac.cn (Y.L.); 2 Yunnan Academy of Forestry and Grassland, Kunming 650201, China; jxcao627@163.com; 3 University of Chinese Academy of Sciences, Beijing 100049, China

**Keywords:** CO_2_ assimilation, mesophyll conductance, photosynthesis, stomatal conductance, photorespiration

## Abstract

Mesophyll conductance to CO_2_ (*g*_m_) act as a significant limiting factor influencing the CO_2_ assimilation rate (*A*_N_) during photosynthetic induction. However, the effect of vapor pressure deficit (VPD) on *g*_m_ kinetics during light induction is not well clarified. We combined gas exchange with chlorophyll fluorescence measurements to assess the induction kinetics of *g*_m_ during light induction under contrasting vapor pressure deficit (VPD) in two tree species with different stomatal conductance (*g*_s_) behavior, *Catalpa fargesii* and *Pterocarya stenoptera*. Our results revealed three key findings: (1) the coordination of *g*_m_ and *g*_s_ kinetics during light induction occurred in *C. fargesii* but not in *P. stenoptera*, and the model of *g*_s_ kinetics largely determines whether the coordination of *g*_s_ and *g*_m_ exist in a given species; (2) a high VPD induced simultaneous changes in *g*_s_ and *g*_m_ kinetics in *C. fargesii* but had separated effects on *g*_s_ and *g*_m_ kinetics in *P. stenoptera*, indicating that the response of *g*_m_ kinetics during light induction to VPD differs between species; and (3) the relative contribution of photorespiration to total electron flow was flexible in response to the change in relative diffusional and biochemical limitations, pointing out that photorespiration has a significant role in the regulation of photosynthetic electron flow during light induction. These results provide new sight into the species-dependent kinetics of *g*_m_ and photorespiration during light induction.

## 1. Introduction

In natural environments, plants experience rapid fluctuations in light intensity on their leaves due to various factors such as shading by neighboring vegetation, cloud cover, and wind-induced changes in leaf orientation. These fluctuations can occur within seconds to minutes and significantly impact photosynthetic processes [1,2,3]. When plants transition from shade to direct sunlight, photosynthesis enters an induction phase, characterized by a gradual increase in the rate of photosynthesis until a new steady state is achieved [4,5,6]. The duration of this induction period, which can range from a few minutes to several tens of minutes, is influenced by plant species, prior light exposure, and environmental conditions [7]. This dynamic response in photosynthesis is crucial for carbon fixation and, ultimately, plant growth [8,9,10,11].

Many previous studies have demonstrated that in fluctuating light environments, the induction phase of photosynthesis can significantly reduce daily carbon assimilation in many crop species compared to an ideal scenario where photosynthetic rates achieve a steady state immediately following a change in light intensity [3,8,12,13]. This highlights the critical importance of understanding the physiological mechanisms underlying photosynthetic induction to potentially improve crop yield [14,15,16,17,18]. The induction process, particularly following a transition from low to high light, is primarily governed by three key physiological processes: the induction rate of photosynthetic electron transport in the thylakoid membrane, the activation of Calvin–Benson cycle enzymes (notably Rubisco), and CO_2_ diffusion conductance.

Photosynthetic electron transport induction is a rapid process that significantly limits photosynthetic efficiency only within the first 1–2 min after the onset of light induction [19]. In contrast, Rubisco activity limits photosynthetic efficiency over a longer period, as it takes 5–20 min for Rubisco to reach its maximum activity level after illumination [20,21]. CO_2_ diffusion conductance involves two sequential components along the CO_2_ diffusion pathway: stomatal conductance (*g*_s_) from the leaf surface to the intercellular spaces and mesophyll conductance (*g*_m_), from the intercellular spaces to the carboxylation sites within the chloroplasts [22]. Over the past decade, research has focused on stomatal kinetics and their impact on photosynthetic induction, revealing significant variations in stomatal opening speeds among and within species [5,23,24,25]. Stomatal conductance typically takes much longer to reach a steady state (from tens of minutes to over an hour) compared to the activation of Rubisco [26,27,28,29]. The slow induction of g_s_ imposes a significant limitation on photosynthesis in some crops, such as African cassava germplasm and tomato species [17].

Compared to the well-studied stomatal limitation, the impact of *g*_m_ on photosynthetic induction is less understood. Studies measuring *g*_m_ at different light levels suggest that *g*_m_ generally increases in response to short-term irradiance increases in a range of plant species, with some exceptions [25,30,31,32,33,34]. However, the response of *g*_m_ to a light shift is not equivalent to its induction kinetics. Only a few studies have attempted to track the time course of *g*_m_ during light induction in model herbaceous species such as Arabidopsis [4,20], tobacco [4,20], and tomato [35,36,37]. These results indicated that the full induction of *g*_m_ requires approximately 7–20 min, which imposes a significant limitation on photosynthesis during light induction. In contrast, the nonsteady-state kinetics of *g*_m_ in response to light induction remains poorly understood in tree species.

A recent study indicated that in the “open stomata” mutant (*ost1*) of Arabidopsis, enhanced *g*_s_ during light induction was accompanied by a more rapid induction rate of *g*_m_, leading to higher photosynthetic efficiency under fluctuating light [4]. Conversely, tomato plants with nitrogen deficiency exhibited simultaneous delays in the induction speeds of both g_s_ and *g*_m_ after transitioning from low to high light [37]. Under drought stress, the decrease in *g*_s_ is associated with a slower induction rate of *g*_m_ in tomato plants, thereby restricting photosynthetic efficiency during light induction [35,36]. These results suggest that during light induction, the kinetics of *g*_s_ significantly influence the induction speed of *g*_m_. However, the hypothesis that *g*_s_ and *g*_m_ kinetics are coordinated during light induction requires further investigation.

Fluctuating light is often accompanied by variations in vapor pressure deficit (VPD) due to diurnal changes in air temperature and relative humidity. VPD is an important environmental factor that affects steady-state photosynthesis primarily by influencing *g*_s_ [38,39,40]. Additionally, high VPD conditions can restrict photosynthesis under fluctuating light by decreasing both the absolute value and the induction rate of *g*_s_ [40,41]. Some studies have reported that elevated VPD induces simultaneous decreases in *g*_s_ and *g*_m_ under fluctuating light in species such as tomato and rose [40,41]. However, the effects of changing VPD on *g*_m_ kinetics in tree species remain poorly understood. Moreover, it is unclear whether the coordination of *g*_s_ and *g*_m_ kinetics during light induction can be altered by changes in VPD. Future research should focus on elucidating these interactions to better understand how VPD influences photosynthetic efficiency under dynamic light conditions.

Within the first minutes after transitioning from low to high light, relatively low diffusional conductance results in a reduced chloroplast CO_2_ concentration [20]. This, in turn, leads to a decreased rate of CO_2_ assimilation, which induces a decline in the ADP/ATP ratio. The reduced ADP/ATP ratio can inactivate chloroplast ATP synthase [42]. Concurrently, the low chloroplast CO_2_ concentration makes photorespiration a significant primary metabolic pathway. Photorespiration consumes a substantial fraction of ATP and NADPH, thereby preventing the feedback inhibition of chloroplast ATP synthase [43]. Therefore, photorespiration may play a crucial role in regulating the photosynthetic electron transport rate during light induction [44]. While the response of photorespiration to light intensity has been studied at a steady state, few investigations have examined the temporal dynamics of photorespiration following an immediate transition from low to high light [45,46].

In the present study, we combined measurements of gas exchange and chlorophyll fluorescence to estimate the kinetics of *g*_m_ during light induction in two tree species with different *g*_s_ behaviors. The main aims were to (1) examine whether the coordination of *g*_s_ and *g*_m_ during light induction is affected by *g*_s_ behavior; (2) characterize the effect of VPD on *g*_m_ kinetics during photosynthetic induction; and (3) quantify the relationship between photorespiration and *g*_m_ kinetics during light induction.

## 2. Results

### 2.1. The Response of The Induction Kinetics of CO_2_ Assimilation, g_s_ and g_m_ to VPD

**Figure 1:**
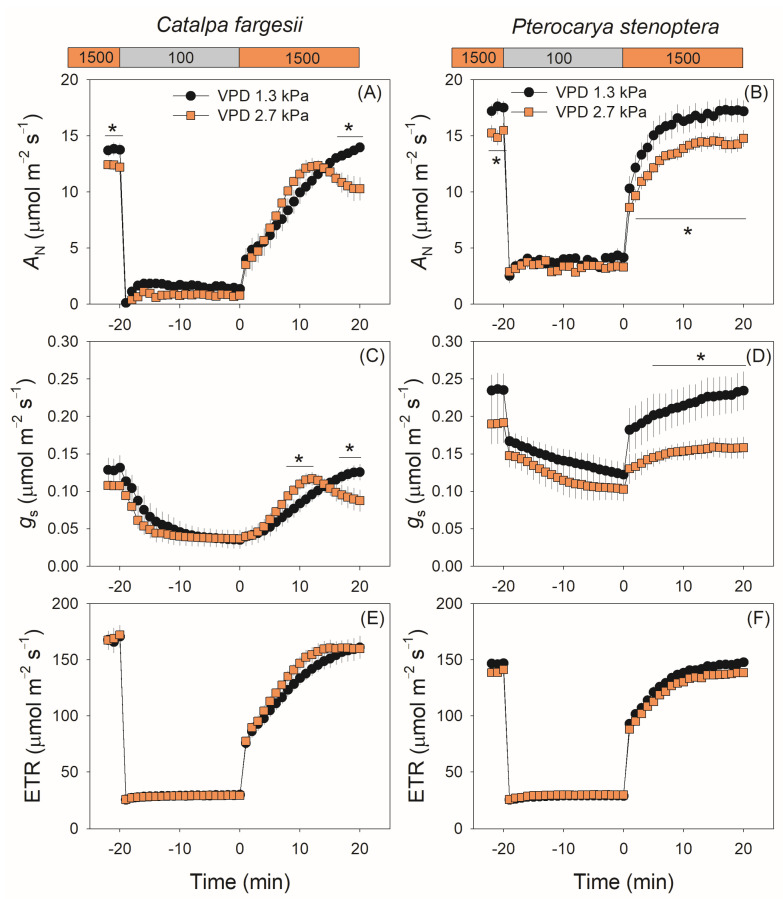
Dynamic change in net CO_2_ assimilation rate (*A*_N_), stomatal conductance (*g*_s_), and electron transport rate (ETR) under fluctuating light for leaves of *Catalpa fargesii* (**A**,**C**,**E**) and *Pterocarya stenoptera* (**B**,**D**,**F**). Fluctuating light alternates between 1500 and 100 µmol m^−2^ s^−1^ every 20 min at 25 °C at different vapor pressure conditions (1.3 and 2.7 kPa). Data are means ± SE (*n* = 6). Asterisk indicates a significant difference between 1.3 and 2.7 kPa VPD conditions (Tukey comparison test, *p* < 0.05).

We first measured the *g*_s_ kinetics in fluctuating light at a moderate VPD of 1.3 kPa. Upon the transfer from high to low light, *g*_s_ gradually decreased in *C. fargesii* with an exponential decline model (Figure 1A). By comparison, in the other species *P. stenoptera*, *g*_s_ largely decreased in the first minute and then gradually decreased with a linear model (Figure 1B). After the transition from low to high light, *g*_s_ gradually increased in *C. fargesii* with a model of exponential rise to the maximum (Figure 1A), whereas *g*_s_ largely increased in the first minute and then gradually increased with a linear model (Figure 1B). Therefore, these two tree species showed different *g*_s_ kinetics model in fluctuating light.

When the vapor pressure deficit (VPD) increased from 1.3 to 2.7 kPa, the *g*_s_ kinetics during light induction were altered in both species. In *C. fargesii*, *g*_s_ first increased to a peak in 10 min and then gradually decreased to a lower steady-state value. By comparison, in *P. stenoptera* the absolute value of *g*_s_ was depressed over time. The kinetics of the net CO_2_ assimilation rate (*A*_N_) showed similar trends to *g*_s_ in both species (Figure 1C,D). In *C. fargesii*, *A*_N_ gradually increased to the steady-state value at 1.3 kPa VPD but first increased to a peak and then gradually decreased at 2.7 kPa VPD, leading to a lower steady-state *A*_N_ at 2.7 kPa than at 1.3 kPa. In *P. stenoptera*, *A*_N_ gradually increased to the steady-state value during light induction, and the absolute value was substantially depressed by the high VPD condition. However, the increased VPD condition had no significant effect on photosynthetic electron transport rate (ETR) during light induction (Figure 1E,F).

**Figure 2:**
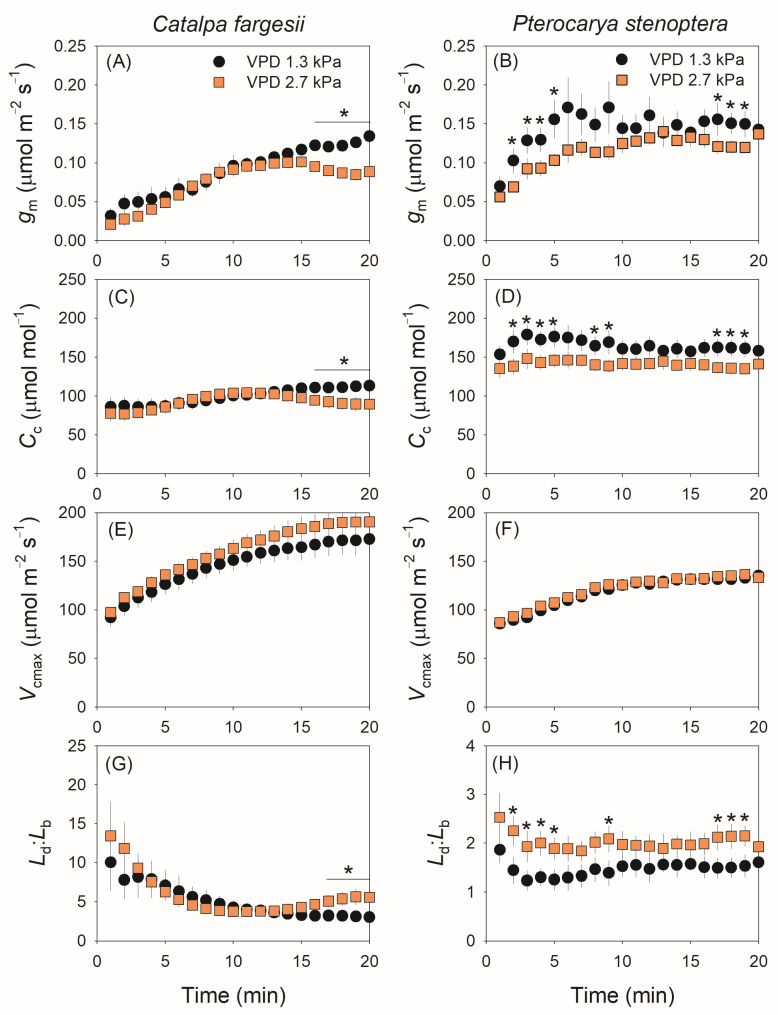
The dynamic change in mesophyll conductance (*g*_m_) and chloroplast CO_2_ concentration (*C*_c_). The maximum carboxylation rate of Rubisco (*V*_cmax_), and the ratio between diffusional and biochemical photosynthesis limitations (*L*_d_:*L*_b_) under fluctuating light for leaves of *Catalpa fargesii* (**A**,**C**,**E**,**G**) and *Pterocarya stenoptera* (**B**,**D**,**F**,**H**). Fluctuating light alternates between 1500 and 100 µmol m^−2^ s^−1^ every 20 min at 25 °C at different vapor pressure conditions (1.3 and 2.7 kPa). Data are means ± SE (*n* = 6). Asterisk indicates a significant difference between 1.3 and 2.7 kPa VPD conditions (Tukey comparison test, *p* < 0.05).

During light induction, the kinetics of *g*_m_ was altered by the high vapor pressure deficit in both species. In *C. fargesii*, *g*_m_ gradually increased in the light induction period (20 min) at 1.3 kPa VPD but first increased to a peak in 15 min and then slightly decreased at 2.7 kPa VPD (Figure 2A). In *P. stenoptera*, *g*_m_ gradually increased to its peak in approximately 6 and 13 min at 1.3 and 2.7 kPa VPD conditions, respectively, and then remained stable (Figure 2B). Compared with 1.3 kPa VPD, the steady-state value of *g*_m_ at 2.7 kPa VPD was depressed in *C. fargesii* but remained stable in *P. stenoptera* (Figure 2A,B). The kinetics of chloroplast CO_2_ concentration (*C*_c_) in light induction showed a similar response to VPD as well as *g*_m_ (Figure 2C,D). By comparison, the rise in VPD hardly affected the kinetics of the Rubisco carboxylation rate (*V*_cmax_) in both species (Figure 2E,F). Owing to the different effects of VPD on CO_2_ diffusion and *V*_cmax_, the ratio between diffusional and biochemical photosynthesis limitations (*L*_d_:*L*_b_) at 2.7 kPa VPD substantially increased at a steady state in *C. fargesii* (Figure 2G) and increased in *P. stenoptera* during light induction (Figure 2H).

### 2.2. Correlation Between g_s_ and g_m_ During Light Induction

**Figure 3:**
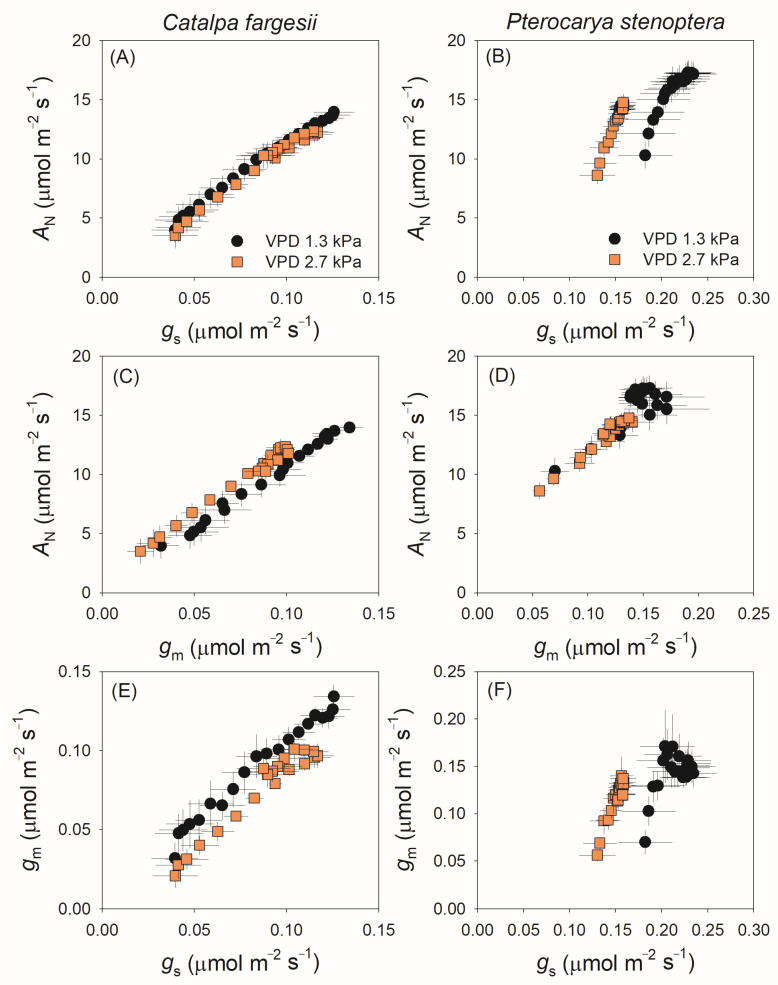
Relationship between net CO_2_ assimilation rate (*A*_N_), stomatal conductance (*g*_s_), and mesophyll conductance (*g*_m_) during light induction for leaves of *Catalpa fargesii* (**A**,**C**,**E**) and *Pterocarya stenoptera* (**B**,**D**,**F**). Data are means ± SE (*n* = 6).

The relationships between *g*_s_, *g*_m_ and *A*_N_ during the induction period are examined in Figure 3. In *C. fargesii*, *A*_N_ was positively correlated to *g*_s_ and *g*_m_ (Figure 3A,C), and a coordination between *g*_s_ and *g*_m_ was observed (Figure 3E). In *P. stenoptera*, the relationship between *A*_N_ and *g*_s_ differed between 1.3 and 2.7 kPa VPD conditions and the same *A*_N_ was accompanied by a much lower *g*_s_ at 2.7 kPa VPD (Figure 3B). Similarly to *C. fargesii*, *A*_N_ was tightly related to *g*_m_ in *P. stenoptera*, especially at 2.7 kPa VPD (Figure 3D). The coordination between *g*_s_ and *g*_m_ in *P. stenoptera* was observed at 2.7 kPa VPD but disappeared at 1.3 kPa VPD, suggesting that the induction of *g*_m_ was independent of *g*_s_ in *P. stenoptera*.

### 2.3. The Time-Integrated Limitations of A_N_ During Light Induction

**Figure 4:**
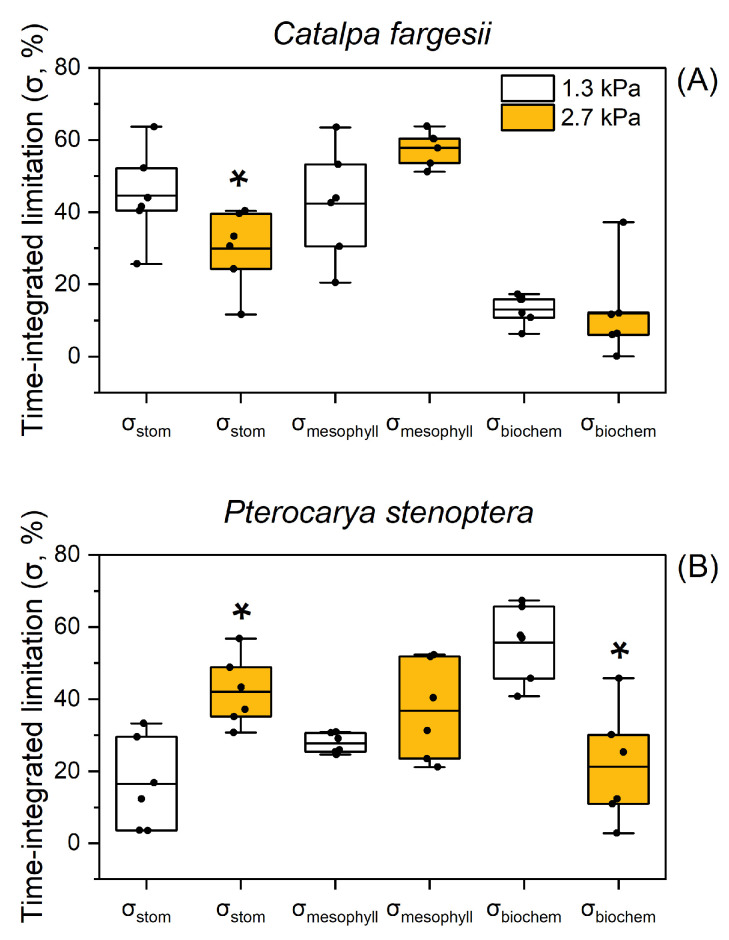
The time-integrated relative limitations by *g*_s_ (σ_stom_), *g*_m_ (σ_mesophyll_) and *V*_cmax_ (σ_biochem_) throughout photosynthetic induction for leaves of *Catalpa fargesii* (**A**) and *Pterocarya stenoptera* (**B**). Data are means ± SE (*n* = 6). The two extreme lines of the boxplot (*whiskers*) show the 10 and 90% percentiles, the two bounds of the box the 25 and 75% percentiles, and the center thick line the median. Dots represent independent data. The asterisk indicates a significant difference between 1.3 and 2.7 kPa VPD conditions (Tukey comparison test, *p* < 0.05).

At 60% relative humidity, the time-integrated limitations of *g*_s_ (σ_stom_), *g*_m_ (σ_mesophyll_), and biochemistry (σ_biochem_) during light induction were 45%, 42%, and 13%, respectively, in *C. fargesii* (Figure 4A). When the atmospheric VPD increased from 1.3 to 2.7 kPa, σ_stom_, σ_mesophyll_, and σ_biochem_ changed to 30%, 58%, and 12%, respectively (Figure 4A). Therefore, CO_2_ diffusional conductance imposed a major limitation on *A*_N_ during light induction in *C. fargesii*. In *P. stenoptera*, the values for σ_stom_, σ_mesophyll_, and σ_biochem_ at 1.3 kPa VPD were 17%, 28%, and 56%, respectively; and they changed to 42%, 37%, and 21% at 2.7 kPa VPD, respectively. This result suggests that a high VPD shifted the major limitation of *A*_N_ from biochemistry to CO_2_ diffusional conductance in *P. stenoptera*.

### 2.4. Induction Kinetics of Photorespiration

**Figure 5:**
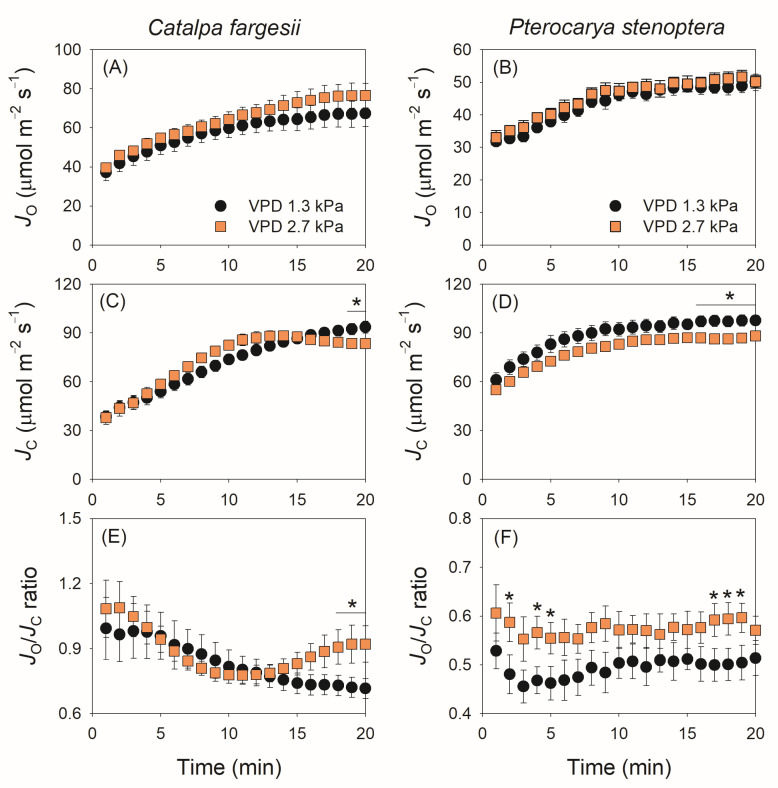
Dynamic change in electron flow for Rubisco oxygenation (*J*_O_) and carboxylation (*J*_C_), and *J*_O_/*J*_C_ ratio during light induction for leaves of *Catalpa fargesii* (**A**,**C**,**E**) and *Pterocarya stenoptera* (**B**,**D**,**F**). Data are means ± SE (*n* = 6). Asterisk indicates a significant difference between 1.3 and 2.7 kPa VPD conditions (Tukey comparison test, *p* < 0.05).

**Figure 6:**
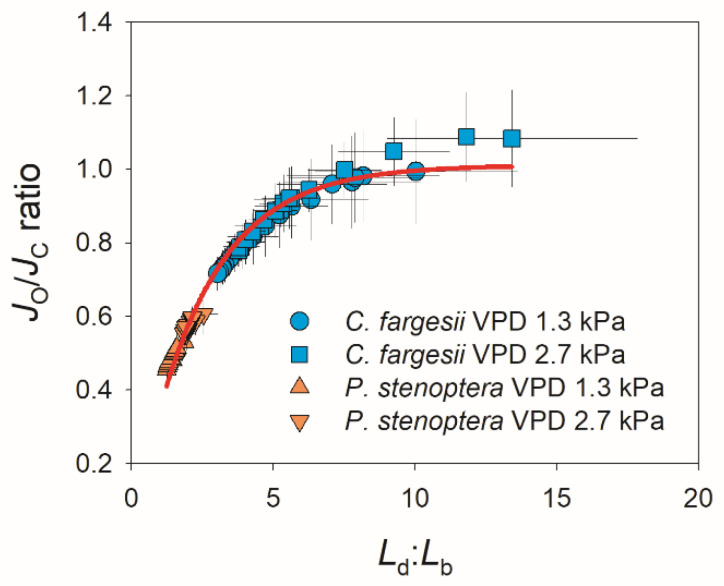
Relationship between *L*_d_:*L*_b_ and *J*_O_/*J*_C_ ratio during light induction for leaves of *Catalpa fargesii* and *Pterocarya stenoptera*. Data are means ± SE (*n* = 6).

The kinetics of photorespiration is shown in Figure 5. Photosynthetic electron flow to Rubisco oxygenation (*J*_O_) gradually increased during light induction in both species, and the changing VPD did not significantly influence the kinetics of *J*_O_ in them (Figure 5A,B). Similarly, the changing VPD slightly affected the kinetics of photosynthetic electron flow to Rubisco carboxylation (*J*_C_). In *C. fargesii*, *J*_C_ gradually increased during the induction period (20 min) at 1.3 kPa VPD but reached the maximum in 12 min at 2.7 kPa VPD (Figure 5C). In *P. stenoptera*, the changing VPD condition did not alter the increase trend in *J*_C_ but decreased the absolute value (Figure 5D). Owing to the different effects of changing VPD on *J*_O_ and *J*_C_ in *C. fargesii,* the *J*_O_/*J*_C_ ratio gradually decreased at 1.3 kPa VPD but first decreased and then gradually increased at 2.7 kPa VPD (Figure 5E). As a result, after light induction for 20 min, the *J*_O_/*J*_C_ ratio at 2.7 kPa VPD was substantially higher than that at 1.3 kPa VPD (Figure 5E). By comparison, in *P. stenoptera,* the *J*_O_/*J*_C_ ratio remained stable at 0.5 and 0.6 during light induction under 1.3 and 2.7 kPa VPD conditions, respectively (Figure 5F). Plotting the data of *J*_O_/*J*_C_ ratio and *L*_d_:*L*_b_ indicated that a non-linear positive relationship was found (Figure 6). When *L*_d_:*L*_b_ was extremely high within the first seconds after the transition to high light, photorespiration acted as a major electron sink to favor the operation of photosynthetic electron flow. When *L*_d_:*L*_b_ was lowered at the later phase of light induction, the contribution of photorespiration to the total electron flow was diminished. Therefore, electron flow to photorespiration is flexible according to the change in *L*_d_:*L*_b_.

## 3. Discussion

### 3.1. Relationship Between g_s_ and g_m_ During Light Induction Is Species-Dependent

During light induction, *g*_s_ gradually increases and imposes significant limitations on photosynthesis in model species such as *Arabidopsis thaliana*, tobacco, tomato and Aferican cassava [4,17,20,35,36]. A few studies have investigated the dynamic kinetics of *g*_m_ during light induction; the results of these studies proposed a hypothesis that *g*_s_ and *g*_m_ coordinated to optimize photosynthesis during light induction [4,20]. We found that the tree species *C. fargesii* exhibited a fine coordination between *g*_s_ and *g*_m_ kinetics during light induction (Figure 3E). However, such a coordination between *g*_s_ and *g*_m_ disappeared in the other tree species *P. stenoptera* (Figure 3F), suggesting that the induction kinetics of *g*_s_ is independent of that of *g*_m_ in *P. stenoptera*. Therefore, coordination between *g*_s_ and *g*_m_ kinetics during light induction is not universal in angiosperms.

After the transition from low to high light, all studied angiosperms showed a similar trend of *g*_m_ kinetics with a model of exponential increase to the maximum, as shown in *Arabidopsis thaliana* [4,20], tobacco [4,20], tomato [35], and the studied tree species *C. fargesii* and *P. stenoptera* (Figure 2A,B). However, the model of *g*_s_ kinetics during light induction varied between species. Generally, there are three different models of *g*_s_ kinetics during light induction among angiosperms: (1) an exponential increase to the maximum, as shown in *Arabidopsis thaliana* [4,20], tobacco [20], and the studied tree species *C. fargesii* (Figure 1C), (2) a sigmoidal increase to the maximum, as shown in tomato [35,37], and (3) a rapid increase in the first minute and then a gradual increase with a linear model, as shown in the studied tree species *P. stenoptera* (Figure 1D). Apparently, in *P. stenoptera,* the different induction models of *g*_s_ and *g*_m_ led to the discoordination between gs and gm during light induction. Therefore, the model of *g*_s_ kinetics largely determines whether the coordination of *g*_s_ and *g*_m_ exist in a given species.

### 3.2. Differential Effects of VPD on g_m_ Kinetics During Light Induction

A high VPD can delay the induction rate of photosynthetic CO_2_ assimilation. One explanation is that a high VPD slows the induction rate of *g*_s_ [5,40,41], but the effect of VPD on *g*_m_ kinetics during light induction is not well understood [41]. Here, we found that the response of *g*_m_ kinetics to VPD was similar to that of *g*_s_ in *C. fargesii* (Figure 1C and Figure 2A). In detail, under the high VPD condition, *g*_m_ first gradually increased to its peak and then gradually decreased to the steady state, with a significantly lower steady-state value than that under the low VPD condition (Figure 2A). By comparison, a high VPD had different effects on the induction kinetics of *g*_s_ and *g*_m_ in *P. stenoptera* (Figure 1D and Figure 2B). Specifically, a high VPD just delayed the induction rate of *g*_m_ but did not significantly affect the steady-state value (Figure 2B). Therefore, the response of *g*_m_ kinetics during light induction to VPD differs between species.

In the tree species *P. stenoptera*, *g*_s_ imposed a minor limitation on *A*_N_ during light induction at a low VPD but acted as a major limitation at a high VPD (Figure 4B). By comparison, the limitation of *g*_m_ imposed on *A*_N_ just increased slightly under the high VPD condition (Figure 4B). Furthermore, the same value of *A*_N_ during light induction was accompanied with a lower *g*_s_ when illuminated at a high VPD (Figure 3B). As a result, the intrinsic water use efficiency (WUEi) was enhanced at a high VPD compared to a low VPD. Concomitantly, the maximum velocity of Rubisco carboxylation (*V*_cmax_) was not altered by the increase in VPD (Figure 2F). Therefore, such an increase in dynamic WUEi under the high VPD condition could not be explained by the change in *V*_cmax_. Alternatively, the dynamic *g*_m_ was slightly affected by the increase in VPD, which compensated for the large decrease in *g*_s_. Consequently, the extent of the decrease in chloroplast CO_2_ concentration (*C*_c_) under the high VPD condition was much lower than that of *g*_s_ (Figure 1D and Figure 2D), which was accompanied by a slight decrease in *A*_N_ (Figure 1B). Therefore, the insusceptibility of *g*_m_ kinetics to VPD has the potential to increase WUEi under high VPD conditions.

### 3.3. Modulation of Photorespiration in Response to Photosynthetic Limitation

Here, we found that within the first few minutes after the transition to high light, photorespiration was a major alternative electron sink in the *C. fargesii*, with the *J*_O_/*J*_C_ ratio being approximately 1.0 (Figure 5E). By comparison, the *J*_O_/*J*_C_ ratio was substantially lower in *P. stenoptera* than in *C. fargesii* (Figure 5E). Therefore, the contribution of photorespiration to total electron flow during light induction significantly differed between *C. fargesii* and *P. stenoptera*. Photorespiration is determined by Rubisco activity and chloroplast CO_2_ concentration (*C*_c_). As shown in Figure 2, the value of *V*_cmax_ was higher in *C. fargesii* than in *P. stenoptera*, while the value of *C*_c_ was lower in *C. fargesii* than in *P. stenoptera*. Therefore, the higher *J*_O_/*J*_C_ in *C. fargesii* than in *P. stenoptera* was mainly caused by their differences in Rubisco activity and chloroplast CO_2_ concentration.

In addition, we found a non-linear positive relationship between the *J*_O_/*J*_C_ ratio and *L*_d_:*L*_b_ in these two studied species (Figure 6), indicating that the relative contribution of photorespiration to total electron flow was flexible in response to the change in relative diffusional and biochemical limitations. When CO_2_ assimilation was limited by the high *L*_d_:*L*_b_ within the first few minutes after the transition from low to high light, the restriction of CO_2_ assimilation increased the ATP/ADP and NADPH/NADP^+^ ratios, rendering chloroplast ATP synthase activity and electron transport from photosystem I to NADP^+^ limited [42]. Photorespiration consumes a significant fraction of ATP and NADPH to maintain the regeneration of RuBP [47] and consequently prevents the feedback inhibition of chloroplast ATP synthase activity and facilitates the operation of the electron downstream of photosystem I [43]. Consistently, photorespiration acted as the major electron sink pathway when *L*_d_:*L*_b_ was extreme high. Alternatively, when CO_2_ assimilation operated efficiently under low *L*_d_:*L*_b_ conditions, the contribution of photorespiration to total electron flow was diminished. Therefore, photorespiration has a significant role in the regulation of photosynthetic electron flow during light induction.

## 4. Materials and Methods

### 4.1. Plant Materials and Growing Conditions

In this study, we used the seedlings of two tree species with different stomatal conductance (*g*_s_) behavior, *Catalpa fargesii* and *Pterocarya stenoptera*. They are deciduous, arboreal trees native to the subtropical regions of southwestern China. The two tree species are light-demanding and typically thrive under full sunlight. Therefore, they have similar light requirements. All plants were cultivated in greenhouses in Kunming, Yunnan, China. The day/night temperature is 30/20 °C, the relative humidity is about 60%, and the maximum light intensity to which leaves are exposed is approximately 1000 μmol photons m^−2^ s^−1^. To avoid any water and nutrient stress, plants were cultivated using a soilless substrate and drip irrigation techniques.

### 4.2. Gas Exchange and Chlorophyll Fluorescence Measurements

Leaves were exposed to high light (1500 µmol photons m^−2^ s^−1^, composed of 90% red light and 10% blue light) using the chlorophyll fluorescence probe of the LI-6400XT portable photosynthesis system for at least 30 min on a sunny summer morning. Steady-state data for gas exchange and chlorophyll fluorescence were then recorded. Subsequently, the light intensity was reduced to 100 µmol photons m^−2^ s^−1^ to simulate a sun-to-shade transition, lasting for 20 min. The light intensity was then returned to 1500 µmol photons m^−2^ s^−1^ to simulate a shade-to-sun transition, also lasting for 20 min. During both sun-to-shade and shade-to-sun transitions, data were recorded at 1-minute intervals. The air temperature was maintained at 25 °C, and the vapor pressure deficit (VPD) was controlled at 1.3 kPa (relative humidity of 60%) and 2.7 kPa (relative humidity of 15%) for different experimental conditions.

In our study, we utilized the multi-phase flash (MPF) protocol following standard procedures to determine the parameters of chlorophyll fluorescence [48]. The light intensity for the measurement was set at 1 µmol m^−2^ s^−1^, while the maximum flash intensity reached 8000 µmol m^−2^ s^−1^. During the second phase of the MPF, the flash intensity was reduced by 60%, with the three flash phases lasting 0.3 s, 0.7 s, and 0.4 s, respectively. Subsequently, we calculated the effective quantum yield of photochemistry for photosystem II (Φ_PSII_) and the total electron transport rate through photosystem II (ETR) using the following equations [49]:

$$\Phi_{PSII}=\frac{\left({F_{m}}^{\prime }-F_{s}\right)}{{F_{m}}^{\prime }}$$

$$ETR=\Phi_{PSII}\times PPFD\times 0.45$$

where PPFD represents the light intensity and 0.45 represents the proportion of light energy absorbed by leaves allocated to photosystem II [50].

### 4.3. Calculation of the Mesophyll Conductance

Mesophyll conductance (*g*_m_) was calculated by combining the gas exchange data and chlorophyll fluorescence data with the following equation [51]:

$$g_{m}=\frac{A_{N}}{C_{i}-\Gamma^{*}(ETR+8\left(A_{N}+R_{d}\right))/(ETR-4\left(A_{N}+R_{d}\right))}$$

where *A*_N_ represents the net photosynthetic rate, *C*_i_ represents the intercellular CO_2_ concentration, Γ* represents the CO_2_ compensation point in the absence of mitochondrial respiration, and a typical value of 40 μmol mol^−1^ is used; *R*_d_ represents the dark respiration rate measured at night.

The chloroplast CO_2_ concentration (*C*_c_) was calculated as follows [52,53]:

$$C_{c}=C_{i}-\frac{A_{N}}{g_{m}}$$

The maximum carboxylation rate of Rubisco (*V*_cmax_) was calculated as follows [54]:

$$V_{cmax}=\frac{\left(A_{N}+R_{d})(C_{c}+K_{c}(1+{O/K}_{o})\right)}{\left(C_{c}-\Gamma^{*}\right)}$$

where *K*_c_ (404 μmol mol^−1^) and *K*_o_ (278 mmol mol^−1^) are the Rubisco Michaelis–Menten constants for CO_2_ and oxygen, respectively; *O* (210 mmol mol^−1^) is the oxygen concentration in the chloroplasts.

### 4.4. Quantitative Calculation of Photosynthetic Limitation

The quantitative calculation formula for the photosynthetic limiting factor is as follows [55]:

$$L_{s}=\frac{g_{tot}/g_{s}\times \partial A_{N}/\partial C_{c}}{g_{tot}+\partial A_{N}/\partial C_{c}}$$

$$L_{m}=\frac{g_{tot}/g_{m}\times \partial A_{N}/\partial C_{c}}{g_{tot}+\partial A_{N}/\partial C_{c}}$$

$$L_{b}=\frac{g_{tot}}{g_{tot}+\partial A_{N}/\partial C_{c}}$$

where *L*_s_, *L*_m_, and *L*_b_ represent the degree of the limitation of photosynthesis by *g*_s_, *g*_m_, and biochemical capacity, respectively, and *g*_tot_ represents the overall CO_2_ diffusive conductance, with *g*_tot_ and ∂*A*_N_/∂*C*_c_ calculated as follows, respectively:

$$g_{tot}=\frac{g_{s}\times g_{m}}{g_{s}+g_{m}}$$

$${\partial A}_{N}/{\partial C}_{c}=V_{cmax}\frac{\Gamma^{*}+K_{c}(1+{O/K}_{o})}{{\left(C_{c}+K_{c}(1+{O/K}_{o})\right)}^{2}}$$

The total CO_2_ diffusional limitation (*L*_d_) was calculated as follows:

$$L_{d}=L_{s}+L_{m}$$

The time-integrated relative limitations imposed by *V*_cmax_ (σ_biochem_), *g*_m_ (σ_mesophyll_) and *g*_s_ (σ_stom_) during the 20 min light induction were calculated according to the detailed method described in [4].

### 4.5. Calculation of Electron Flow for Photorespiration

The rate of electron flow for photorespiration (*J*_O_) and Rubisco carboxylation (*J*_C_) were calculated according to the following equations [56]:

$$J_{O}=2/3\times (ETR-4\times (A_{N}+R_{d}))$$

$$J_{C}=1/3\times (ETR+8\times (A_{N}+R_{d}))$$

### 4.6. Statistical Analysis

Six independent leaves from six different plants were used for each measurement. One-way ANOVA (the Tukey comparison test) was used to determine whether significant differences (*α* = 0.05) existed between the low and high VPD treatments. The software SigmaPlot 10.0 was used for graphing and fitting.

## 5. Conclusions

Our findings indicate that the coordination of *g*_s_ and *g*_m_ during light induction is determined by *g*_s_ kinetics. The effect of VPD on *g*_m_ kinetics during light induction is species-dependent. Concomitantly, the flexibility of photorespiration plays an important role in the regulation of photosynthetic electron flow in response to the kinetics of *g*_m_. Therefore, we call for more studies to investigate the role of photorespiration during light induction with the change in *g*_m_ kinetics under environmental stresses.

## Data Availability

The raw data supporting the conclusions of this article will be made available by the authors on request.
